# Kazakh national dog breed Tazy: What do we know?

**DOI:** 10.1371/journal.pone.0282041

**Published:** 2023-03-08

**Authors:** Anastassiya Perfilyeva, Kira Bespalova, Sergey Bespalov, Мamura Begmanova, Yelena Kuzovleva, Zhassulan Zhaniyazov, Olga Vishnyakova, Inna Nazarenko, Yuliya Perfilyeva, Ozada Khamdiyeva, Bakhytzhan Bekmanov

**Affiliations:** 1 Department of Molecular Genetics, Institute of Genetics and Physiology, Almaty, Kazakhstan; 2 Department of Biology and Biotechnology, Al-Farabi Kazakh National University, Almaty, Kazakhstan; 3 Department of Theriology, Institute of Zoology, Almaty, Kazakhstan; 4 Department of Сynology, Republican Federation of Public Associations of Hunters and Hunting Societies "Kansonar", Almaty, Kazakhstan; 5 Department of Сynology, Republican Federation of Public Associations of Hunters and Hunting Societies "Kansonar", Astana, Kazakhstan; 6 Department of Immunology, M.A. Aitkhozhin’s Institute of Molecular Biology and Biochemistry, Almaty, Kazakhstan; Institute of Mediterranean Forest Ecosystems of Athens, GREECE

## Abstract

The Tazy or Kazakh National sighthound has been officially recognized as the national heritage of Kazakhstan. Comprehensive genetic studies of genetic diversity and population structure that could be used for selection and conservation of this unique dog breed have not been conducted so far. The aim of this study was to determine the genetic structure of the Tazy using microsatellite and SNP markers and to place the breed in the context of the world sighthound breeds. Our results showed that all 19 microsatellite loci examined were polymorphic. The observed number of alleles in the Tazy population varied from 6 (INU030 locus) to 12 (AHT137, REN169D01, AHTh260, AHT121, and FH2054 loci) with a mean of 9.778 alleles per locus. The mean number of effective alleles was 4.869 and ranged from 3.349 f to 4.841. All markers were highly informative (PIC values greater than 0.5) and ranged from 0.543 (REN247M23 locus) to 0.865 (AHT121 locus). The observed and expected heterozygosities in a total population were 0.748 and 0.769 and ranged from 0.746 to 0.750 and 0.656 to 0.769, respectively. Overall, the results confirmed that the Tazy breed has a high level of genetic diversity, no significant inbreeding, and a specific genetic structure. Three gene pools underlie the genetic diversity of the Tazy breed. SNP analysis using the CanineHD SNP array, which contains more than 170,000 SNP markers, showed that the Tazy breed is distinct from other sighthound breeds and genetically related to ancient eastern sighthound breeds sharing the same branch with the Afghan Hound and the Saluki. The results, together with archeological findings, confirm the ancient origin of the breed. The findings can be used for the conservation and international registration of the Tazy dog breed.

## Introduction

The Tazy is a breed of sighthound common in Kazakhstan. Kazakhs hunted hares, wild boars, foxes, badgers, deer, wolves and saigas with the Tazy since ancient times. The role of the Tazy is well documented by famous travelers and explorers of the 18th century such as Pallas P. S., Falk I. P., and Georgi I. G. The description of the Tazy as a distinct breed was made by Machevarianov P. M. [1], Bogdanov M. [2], Sabaneev L. P. [3], and Sludsky A.A. [4]. Unfortunately, the popularity of the Tazy declined when more modern hunting methods became available. Their population size seems to have gradually decreased in recent decades. Unofficial estimates indicate that there are currently only about 3,000 Tazy dogs left, but no more than 350 high-quality dogs with pedigrees up to the fourth generation. After the resumption of efforts to preserve this unique breed, the Tazy was granted the status of a national dog breed, and the modern standard of the Tazy breed was developed (S1 Table).

Nevertheless, this unique breed is not recognized by any of the major kennel clubs, neither the American Kennel Club (AKC), the United Kennel Club (UKC), the Canadian Kennel Club (CKC), nor the Fédération Cynologique Internationale (FCI) and for most dog breeders in the world, the Tazy is still unknown and confused with the Saluki. In fact, the phenotypic differences between the Tazy and the Saluki are barely perceptible and concern the structure of the coat, the shape of the head and the chest. There is a hypothesis that these breeds and other eastern sighthounds (Sloughi, Azawakh, Afghan Hound, etc.) are descended from a single common ancestor such as the ancient Egyptian Tezem (or Tesem) [3, 5]. However, despite the popularity of this hypothesis, some authors deny that there is evidence that the sighthounds are descended from the Tezem [6]. The Sloughi has been cited as a second likely common ancestor [5], but mitochondrial DNA analysis revealed that the Tazy has no sequences in common with the Sloughi but does have sequences in common with the Saluki [7]. This agrees well with the hypothesis that the Tazy probably originated from a cross between the Saluki and the Mongolian Shepherd [5], which is supported by breed cluster data based on SNP markers showing that the Saluki is one of the oldest breeds in the world [8–10]. An alternative hypothesis is that the ancestors of sighthounds were the Tazy or Hortaya Borzaya, since Kazakhstan and the Don and Dnieper steppes are possible centers for the domestication of the horse [11]. It is known that the first sighthounds may be found in those ancient cultures where the hunter is a horseman, archer, and nomad, i.e., in cultures where the horse was domesticated, and tribes moved freely in open landscapes [5]. The origin of the sighthounds was probably closely related to the process of domestication of the horse and the development of horsemanship, which makes Kazakhstan a possible place of origin of the sighthounds [5].

So, the discussion about the origin of the sighthounds and the position of the Tazy among the other breeds continues, and it becomes important to study the genetic structure of the Tazy and to get a picture of how well the breed self-segregates and relates to similar breeds. Microsatellite markers, or Short Tandem Repeat (STR) markers, are a well-known, effective, and powerful tool commonly used to study the genetic structure and diversity of dog breeds [12–15]. Evaluation and monitoring of genetic variability facilitates selection of the optimal mating partner and determination of the degree of inbreeding. Information obtained through microsatellite marker studies is particularly useful for native dog breeds. Recently, SNP-based analysis has opened new possibilities for genetic analysis of dog breeds. A few studies have reported the successful identification of breed structure and evolutionary relationships among dog breeds using high-density SNP microarrays [9, 15–17]. To our knowledge, the genetic diversity and structure of the Tazy dogs has not been studied. The aim of our study was to provide insight into the genetic structure and history of the Tazy using two effective and widely used genetic markers such as STR loci and SNPs. To determine the position of the Tazy among other sighthounds, we characterized the genetic profile of the Tazy by comparing it to recognized sighthound breeds and estimated the genetic differentiation within and between these breeds. The results of the study could be a unique tool for developing strategies for effective management and conservation of the Tazy’s genetic resources and for international recognition of the breed.

## Material and methods

### Sample collection and DNA extraction

All experimental procedures were approved by the Ethics Committee of the Institute of Human and Animal Physiology, Almaty, Kazakhstan (# 3, September 15th, 2020).

DNA samples were obtained by collecting buccal swabs and/or blood samples at dog shows, special events, and mail donations. Between April 2021 and May 2022, a total of 134 samples were collected from six breeds of sighthound: Whippet (n = 5), Russian Borzoi (n = 4), Greyhound (n = 8), Afghan Hound (n = 2), Saluki (n = 1), and Tazy (n = 114). The Tazy dogs were collected in three locations: South Kazakhstan (TSK, n = 58), North Kazakhstan (TNK, n = 46), and East Kazakhstan (TЕK, n = 10). Assessment of compliance with the breed standard was performed by the expert of the national breed affiliated association "Kansonar". All owners gave informed written consent to use samples from their dogs for genetic studies. Detailed pedigree information was requested for all dogs. When pedigree information was available, only minimally related animals were included in the sample. For those animals for which no pedigree was available, non-relatedness was verified by the handlers. DNA was extracted using QiaAmp DNA extraction kits according to the manufacturer’s protocol (Qiagen, Valencia, CA).

### STR genotyping

A total of 19 microsatellite markers (AHTk211, CXX279, REN169O18, INU055, REN54P11, INRA21, AHT137, REN169D01, AHTh260, AHTk253, INU005, INU030, amelogenin, FH2848, AHT121, FH2054, REN162C04 AHTh171, and REN247M23) recommended by ISAG were amplified using a commercial kit Canine ISAG STR Parentage Kit (Thermo Fisher Scientific, CA, USA) for a total of 134 DNA samples. Genotyping was performed using the SeqStudio^™^ Genetic Analyzer (Thermo Fisher Scientific, CA, USA). Microsatellite alleles were processed and manually verified using GeneMapper^™^ Software 6 (Thermo Fisher Scientific, CA, USA).

### SNP genotyping

SNP genotyping using an Illumina Infinium CanineHD Genotyping BeadChip (Illumina Inc. San Diego, CA) was performed for a total of 39 DNAs of the Tazy with the highest expert scores, whose functional health and body characteristics meet the standard for the Tazy breed and are able to work and perform functions according to the specific characteristics of the Tazy breed (S1 Fig). SentrixBarcode and SentrixPosition on the chip are listed in S2 Table. Genotype data have been deposited in an Open Science Framework repository and are available at: DOI 10.17605/OSF.IO/5SHWU.

### Analysis of the STR data

Allele frequencies of 18 STR loci were used to determine average alleles/locus (Na), average effective alleles/locus (Ne), observed heterozygosity (Ho), expected heterozygosity (He), inbreeding coefficient F, and principal coordinate analysis (PCoA) using GenAIEX 6.5 [18–20]. Polymorphism information content (PIC) and deviations from Hardy-Weinberg equilibrium (HWE) were tested using Cervus 3.0.7 [21]. Bonferroni corrections were applied for HWE estimation. Bayesian clustering was performed using Structure v2.3.4 [22, 23]. Simulations were performed assuming a model of admixture and correlated allele frequencies with 10 000 burn-in steps and 100 000 MCMC steps. Ten independent runs were performed for each assumed number of clusters (K), with K varying from 1 to 10. The most likely number of clusters was determined in CLUMPAK25 (http://clumpak.tau.ac.il/index.html) using the method of Evanno et al. [24]. The analysis was performed twice, the first time using the Tazy database and a second time using the database of all sighthound breeds.

### Analysis of the SNP data

The SNP genotype data generated by the iScan system were loaded into the Illumina GenomeStudio program to perform the primary data analysis and generate a final custom report (PED and MAP) for downstream analysis. Data obtained from the 39 Tazy were merged with publicly available SNP array data of 89 dogs from seven sighthound breeds and 14 Gray Wolves downloaded from the Dryad repository (datadryad.org, doi:10.5061/dryad.v9t5h; doi:10.5061/dryad.pm7mt). The sample code and corresponding breed are listed in S3 Table. In the PLINK 1 (www.cog-genomics.org/plink/1.9/) [25] Input Report, 166,171 SNPs of the 89 samples from seven breeds and 14 Gray Wolves and 172,115 SNPs of the 39 samples from the Tazy dogs were filtered using the following steps: (1) removal of very closely related individuals PI_HUT > 0.4; (2) filtering of SNPs that have an exact Hardy-Weinberg equilibrium (—hwe 0.01); (3) removal of SNPs on the X and Y chromosomes (—not-chr); (4) selection of only SNPs with minor allele frequency (—maf) **>** 0.05; (5) calling rate SNP (—geno) 0.05; (6) removal of SNPs with pairwise genotypic associations (r2) > 0.2 within a window of 50 SNPs (—indep-pairwise 50 5 0.2). The number of SNPs retained for calculations after the filtering process was 40,229 autosomal SNPs. The PCoA of unrelated dogs was performed using PLINK 1.9 software and visualized in the R package "ggplot2" [26, 27].

## Results

### Diversity analysis based on the STR dataset

A total of 176 alleles were detected at the 18 microsatellite loci. Table 1 shows the variability parameters of the analyzed loci in a total population of the Tazy and in three subpopulations.

**Table 1:** Polymorphism of 18 STR markers of the Tazy.

Locus	Na				Ne				Ho				He				F			
	TSK	TNK	TЕK	All	TSK	TNK	TЕK	All	TSK	TNK	TЕK	All	TSK	TNK	TЕK	All	TSK	TNK	TЕK	All
AHTk211	6	7	4	7	3.255	2.374	2.778	2.884	0.638	0.543	0.900	0.623	0.693	0.579	0.640	0.653	0.079	0.061	-0.406	0.047
CXX0279	9	7	6	10	4.515	4.089	4.545	4.537	0.810	0.674	0.900	0.763	0.779	0.755	0.780	0.780	-0.041	0.108	-0.154	0.021
REN169O18	6	8	4	8	5.082	5.350	3.509	5.405	0.759	0.848	0.800	0.798	0.803	0.813	0.715	0.815	0.056	-0.043	-0.119	0.021
INU055	8	6	3	9	3.441	3.340	2.174	3.426	0.690	0.696	0.600	0.684	0.709	0.701	0.540	0.708	0.028	0.007	-0.111	0.034
REN54P11	8	9	6	10	5.483	4.301	4.167	5.366	0.879	0.739	1.000	0.833	0.818	0.767	0.760	0.814	-0.075	0.037	-0.316	-0.024
INRA21	9	7	5	10	3.765	3.722	3.448	3.804	0.759	0.783	1.000	0.789	0.734	0.731	0.710	0.737	-0.033	-0.070	-0.408	-0.071
AHT137	11	12	6	12	5.755	5.337	4.082	6.129	0.793	0.826	0.600	0.789	0.826	0.813	0.755	0.837	0.040	-0.017	0.205	0.057
REN169D01	12	10	7	12	8.057	7.222	4.000	7.932	0.914	0.870	1.000	0.904	0.876	0.862	0.750	0.874	-0.043	-0.009	-0.333	-0.034
AHTh260	11	9	4	12	4.087	3.443	2.353	3.846	0.776	0.761	0.700	0.763	0.755	0.710	0.575	0.740	-0.027	-0.072	-0.217	-0.031
AHTk253	7	8	4	8	2.789	3.443	2.041	2.981	0.603	0.587	0.400	0.579	0.641	0.710	0.510	0.665	0.059	0.173	0.216	0.129
INU005	11	7	7	11	4.577	4.793	4.000	4.759	0.759	0.739	1.000	0.772	0.782	0.791	0.750	0.790	0.029	0.066	-0.333	0.023
INU030	6	6	3	6	3.379	3.361	1.504	3.260	0.603	0.696	0.200	0.605	0.704	0.703	0.335	0.693	0.143	0.010	0.403	0.127
FH2848	8	8	5	8	4.782	5.218	3.077	5.027	0.776	0.826	0.700	0.789	0.791	0.808	0.675	0.801	0.019	-0.022	-0.037	0.015
AHT121	12	12	9	12	6.886	8.447	7.407	8.097	0.810	0.935	1.000	0.877	0.855	0.882	0.865	0.877	0.052	-0.060	-0.156	-0.001
FH2054	9	10	5	12	6.253	5.539	4.255	5.961	0.862	0.783	0.800	0.825	0.840	0.819	0.765	0.832	-0.026	0.045	-0.046	0.009
REN162C04	9	9	4	9	4.675	5.317	2.198	4.837	0.793	0.783	0.700	0.781	0.786	0.812	0.545	0.793	-0.009	0.036	-0.284	0.016
AHTh171	10	10	5	11	7.953	5.944	2.632	7.029	0.776	0.783	0.700	0.772	0.874	0.832	0.620	0.858	0.113	0.059	-0.129	0.100
REN247M23	7	7	4	9	2.404	2.375	2.105	2.370	0.483	0.565	0.500	0.518	0.584	0.579	0.525	0.578	0.173	0.024	0.048	0.105
Mean	8.833	8.444	5.056	9.778	4.841	4.645	3.349	4.869	0.749	0.746	0.750	0.748	0.769	0.759	0.656	0.769	0.030	0.018	-0.121	0.030
SE	0,4731	0,4295	0,3658	0,4469	0,3886	0,3757	0,3238	0,3983	0,0256	0,0249	0,0544	0,0247	0,0188	0,0201	0,0309	0,0196	0,0190	0,0203	0,0203	0,0197

Abbreviations: All = Three subpopulations; Na = Average alleles/locus; Ne = Average effective alleles/locus; Ho = Observed heterozygosity; He = Expected heterozygosity; F = Inbreeding coefficient.

The highest number of alleles was detected for AHT137, REN169D01, AHTh260, AHT121, and FH2054 (12 alleles for each locus), and the lowest for INU030 (6 alleles per locus). The average number of alleles per locus was 9.778 and ranged from 5.056 for TEK to 8.833 for TSK. The average number of effective alleles was 4.869 and ranged from 3.349 for TEK to 4.841 for TSK. There were no significant differences in Ho among all three subpopulations (Ho = 0.746–0.750). In contrast, He was highest in TSK (0.769 *vs*. 0. 0.656 in TEK and 0.759 in TNK). The inbreeding coefficient within subpopulations (F) ranged from -0.121 (TEK) to 0.030 (TSK), whereas the overall inbreeding coefficient for all Tazy dogs was 0.030. The mean value of PIC for all Tazy dogs was estimated to be approximately 74%, ranging from 54% for REN247M23 to 87% for AHT121 (Table 2). PIC above 60% was observed for all STR markers except REN247M23, whereas a value above 80% was observed for 5 loci (AHT137, REN169D01, AHT121, FH2054, and AHTh171).

**Table 2:** PIC of 18 STR markers of the Tazy.

Locus	PIC
AHTk211	0.614
CXX0279	0.746
REN169O18	0.789
INU055	0.653
REN54P11	0.789
INRA21	0.702
AHT137	0.819
REN169D01	0.861
AHTh260	0.701
AHTk253	0.609
INU005	0.764
INU030	0.653
FH2848	0.774
AHT121	0.865
FH2054	0.811
REN162C04	0.765
AHTh171	0.842
REN247M23	0.543
Mean	0.739
SE	0.094

Abbreviations: PIC = Polymorphism information content.

The Hardy-Weinberg test performed separately for each locus showed no deviation from the expected frequencies after Bonferroni correction (*P* > 0.05), except for INU030 (*P* < 0.01) (Table 3).

**Table 3:** HWE for 18 STR markers in total population.

Locus	*P* (with Bonferroni correction)	Significance
AHTk211	0.320	NS
CXX0279	0.888	NS
REN169O18	0.868	NS
INU055	0.971	NS
REN54P11	0.952	NS
INRA21	0.083	NS
AHT137	0.694	NS
REN169D01	0.836	NS
AHTh260	0.801	NS
AHTk253	0.090	NS
INU005	0.360	NS
INU030	0.007	S**
FH2848	0.457	NS
AHT121	0.903	NS
FH2054	0.101	NS
REN162C04	0.811	NS
AHTh171	0.229	NS
REN247M23	0.138	NS

Abbreviations: NS = Not significant;

** *P* <0.01,

### Tazy population structure based on the STR dataset

**Fig 1:**
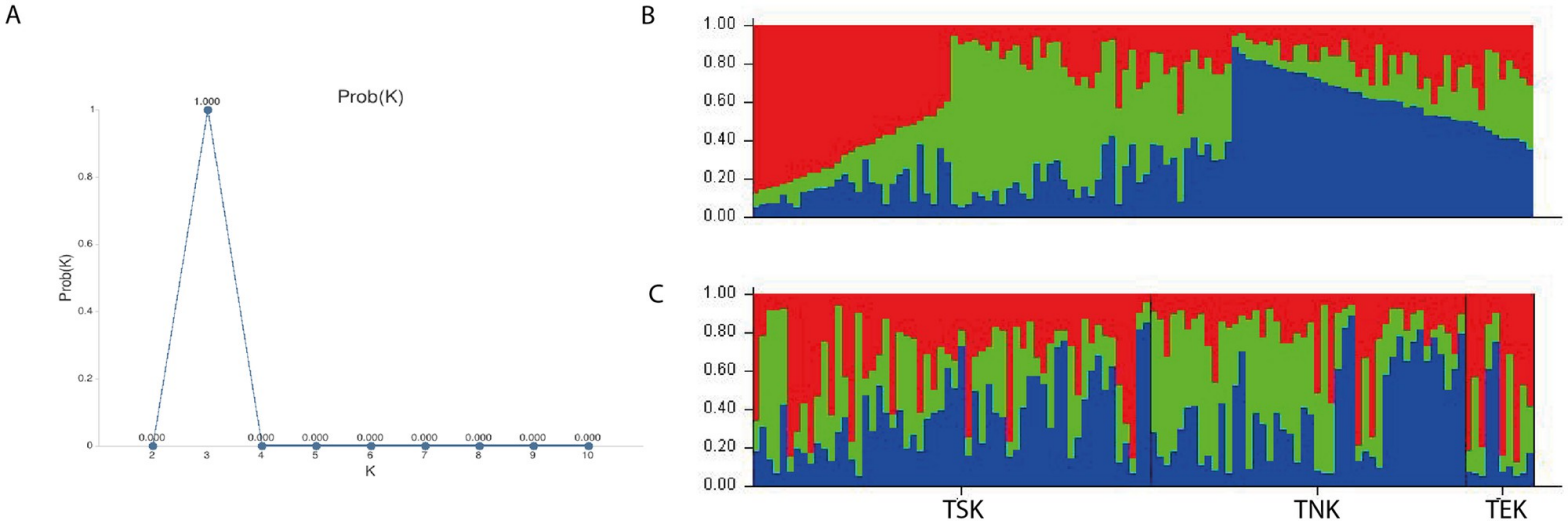
Genetic structure of the Tazy subpopulations. Bayesian clustering on the STR dataset of 114 dogs performed with STRUCTURE v2.3.4 after correction by Evanno et al. (CLUMPAK): A- Delta K results; B and C- bar plots where each dog accession is represented by a single vertical line, and this line shows colored segments representing the relative percentage of membership in the K cluster. B—bar plot grouped by clusters; C—bar plot grouped by subpopulations. Abbreviations of subpopulations: TSK—Tazy of South Kazakhstan, TNK—Tazy of Northern Kazakhstan, TЕK—Tazy of Eastern Kazakhstan.

To further characterize the genetic variation of breed the STRUCTURE analysis was applied (Fig 1). The Delta K results showed that the optimal number of genetic clusters representing the most similar individuals was K = 3 (Fig 1A), indicating that three gene pools shaped the genetic structure of the Tazy population under study (Fig 1B). Based on K = 3, the proportion of each of the three gene clusters is present in each subpopulation (Fig 1C). S4 Table shows the detailed results on the composition of the clusters and the percentage of membership in each accession for three clusters in one run. The distance between each cluster is shown in Table 4. Cluster 3 is very similar to clusters 1 and 2 (0.040 for both), while clusters 1 and 2 differ slightly more (0.082). The average distances (expected heterozygosity) between individuals in the same cluster were found to be highest for cluster 3 (0.808) (Table 4). The estimated mean Fst value for accessions in cluster 1 was 0.164, in cluster 2–0.157, and in cluster 3–0.003.

**Table 4:** Allele-freq. divergence (Net nucleotide distance), computed using point estimates of P through Structure analysis.

Cluster	1	2	3	Average distances	Mean value of Fst
1	-	0.082	0.040	0.689	0.164
2	0.082	-	0.040	0.715	0.157
3	0.040	0.040	-	0.808	0.003

Abbreviations: Fst = Degree of differentiation among populations

**Fig 2:**
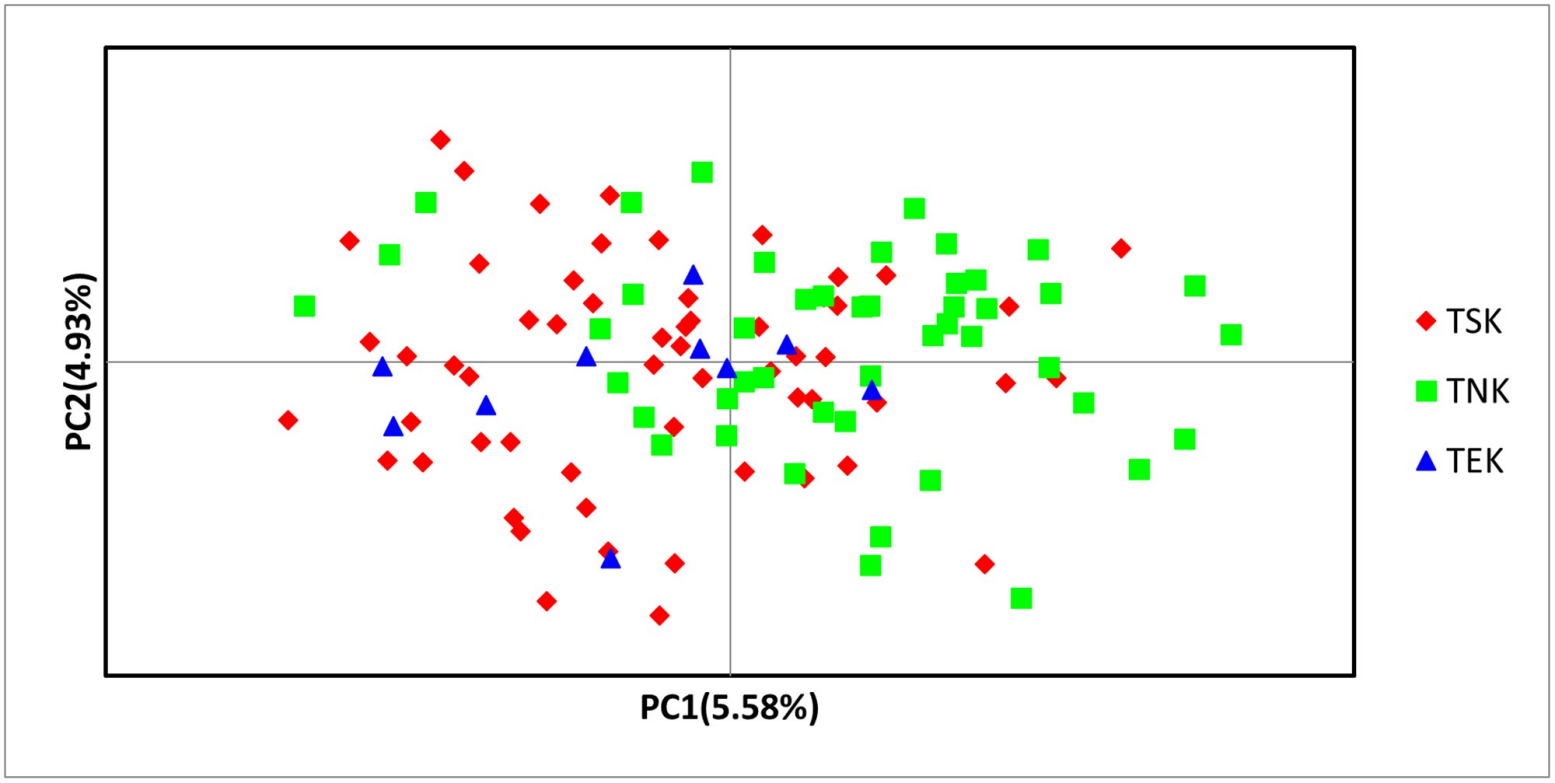
PCoA plot of 114 Tazy dogs from three different subpopulations based on STR data.

The PCoA of the STR data across three subpopulations revealed three significant axes: axis one explained 5.58% of the variation present, axis two 4.93%, and axis three 4.65%. On both axis one and axis two (Fig 2), the TSK and TNK subpopulations had the greatest variation in allelic diversity. All three subpopulations were mixed, confirming the results of Bayesian clustering.

### Breed relationships based on the STR dataset

**Fig 3:**
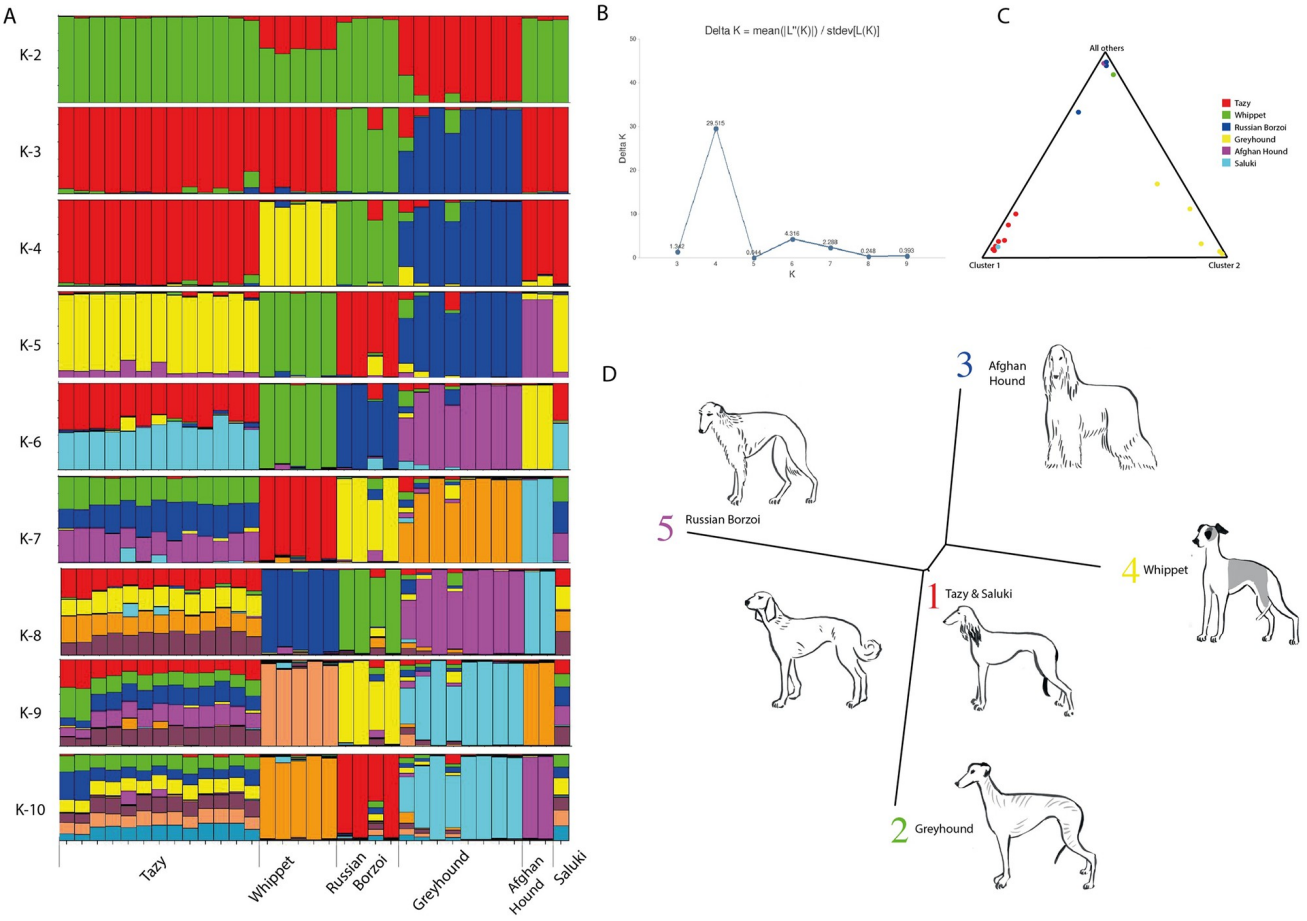
Genetic structure of sighthounds breeds. A—Bar plot. Bayesian clustering on the STR dataset of six sighthounds dogs performed with STRUCTURE v2.3.4 for a priori distinct number of K = 2–10; B—Delta K results; C—triange plot showing distribution of six breeds of sighthound in five clusters; D—tree plot representing the genetic distance between the STRUCTURE clusters. Canine images not drawn to scale. Reprinted from https://drive.google.com/drive/folders/1hFzoHRWnQ7-eNeKDZKwBy_nLaUs3bTJY?usp=share_link] under a CC BY license, with permission from Anna Molodykh, original copyright 2022.

Then we applied STRUCTURE to STR data from the Tazy and other five sighthound breeds to analyze the relationships. Plots for K values from 1 to 10 are shown in Fig 3A. The software identified K = 5 as the most likely number of genetic clusters in our samples (Fig 3B). S5 Table shows the detailed results on the composition of the clusters and the percentage of membership in each accession for five clusters in one run. In the visualized structure of one run at K = 5, the Greyhound, the Afghan Hound, the Whippet, and the Russian Borzoi form distinct clusters consisting of dogs of the respective breeds (Fig 3A). At K = 5, the contribution of the Tazy and the Saluki to the Afghan Hound can be detected, but at K = 6, the Afghan Hound separates and remains relatively stable. The Tazy and the Saluki show similar mixed profiles (Fig 3A). Similarly, in the Structure output presented as a triangle plot (Fig 3C), the Tazy and the Saluki are close together in one corner and are assigned to one cluster. The distance between each cluster is shown in Table 5. The largest distance was found for cluster 2 Greyhound *vs* cluster 5 Russian Borzoi, (0.216), followed by cluster 2 *vs* cluster 3 Afghan Hound (0.197). Cluster 1 Tazy and Saluki was related to clusters 3 and 4 Whippet (0.082 and 0.085, respectively), as shown in the tree plot (Fig 3D). The mean Fst value (Table 5) for the accessions in cluster 1 Tazy and Saluki had the lowest estimated value compared to other breeds (0.003).

**Table 5:** Allele-freq. divergence (Net nucleotide distance), computed using point estimates of P through Structure analysis.

Cluster	1	2	3	4	5	Average distances	Mean value of Fst
1	-	0.107	0.082	0.085	0.110	0.812	0.003
2	0.107	-	0.197	0.192	0.216	0.609	0.280
3	0.082	0.197	-	0.142	0.193	0.628	0.337
4	0.085	0.192	0.142	-	0.195	0.623	0.305
5	0.110	0.216	0.193	0.195	-	0.575	0.354

**Fig 4:**
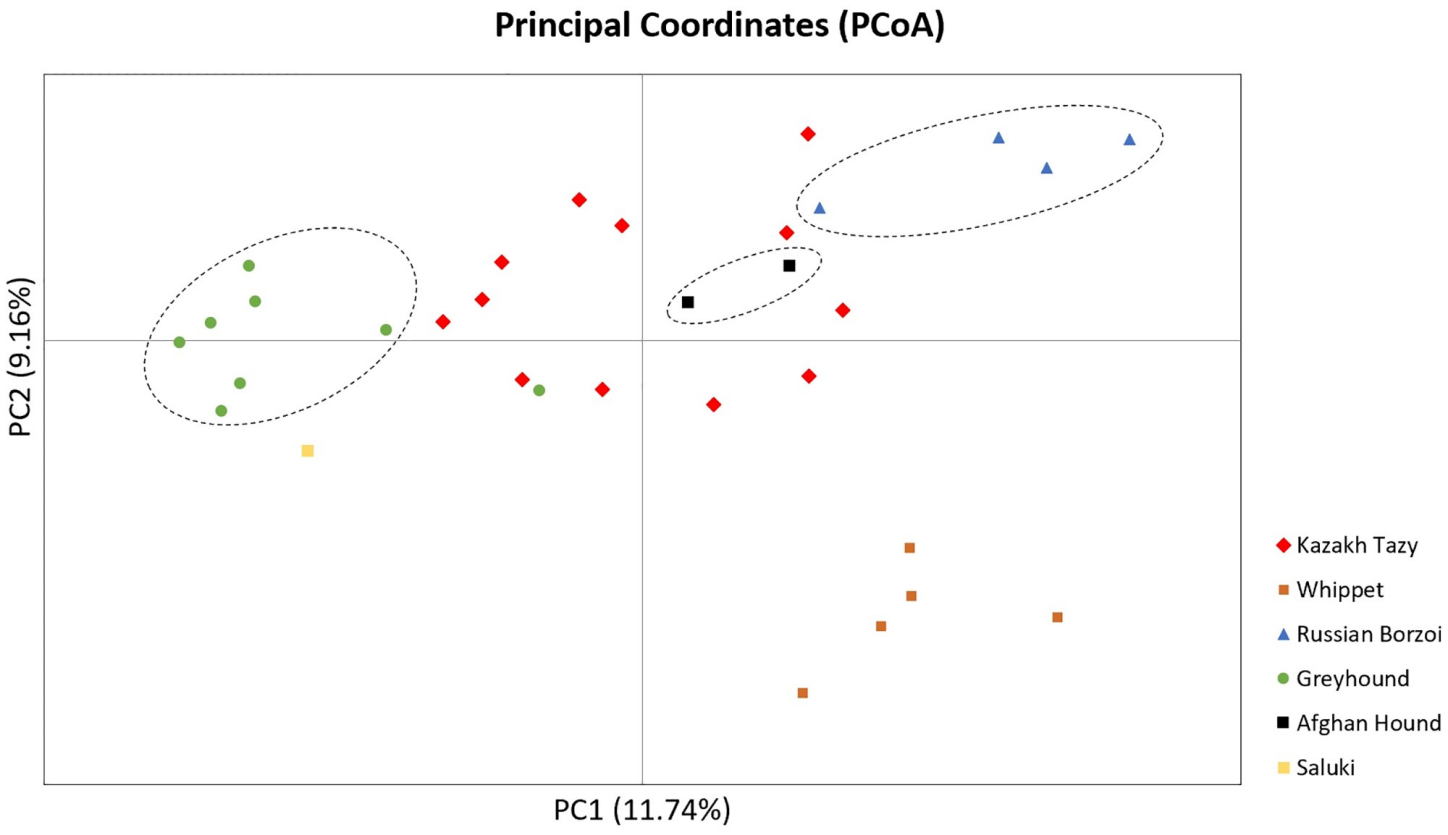
PCoA plot showing population structure of sighthound breeds based on the STR data.

The PCoA (Fig 4), both the first and second axes, which accounted for 11.74% and 9.16% of the total variance, respectively, separated the Saluki from the Tazy dogs. The Tazy and the Afghan Hound were clustered together, which is consistent with the result of the STRUCTURE analysis, in which the relatedness of these two breeds were indicated.

### Breed relationships based on the SNP dataset

**Fig 5:**
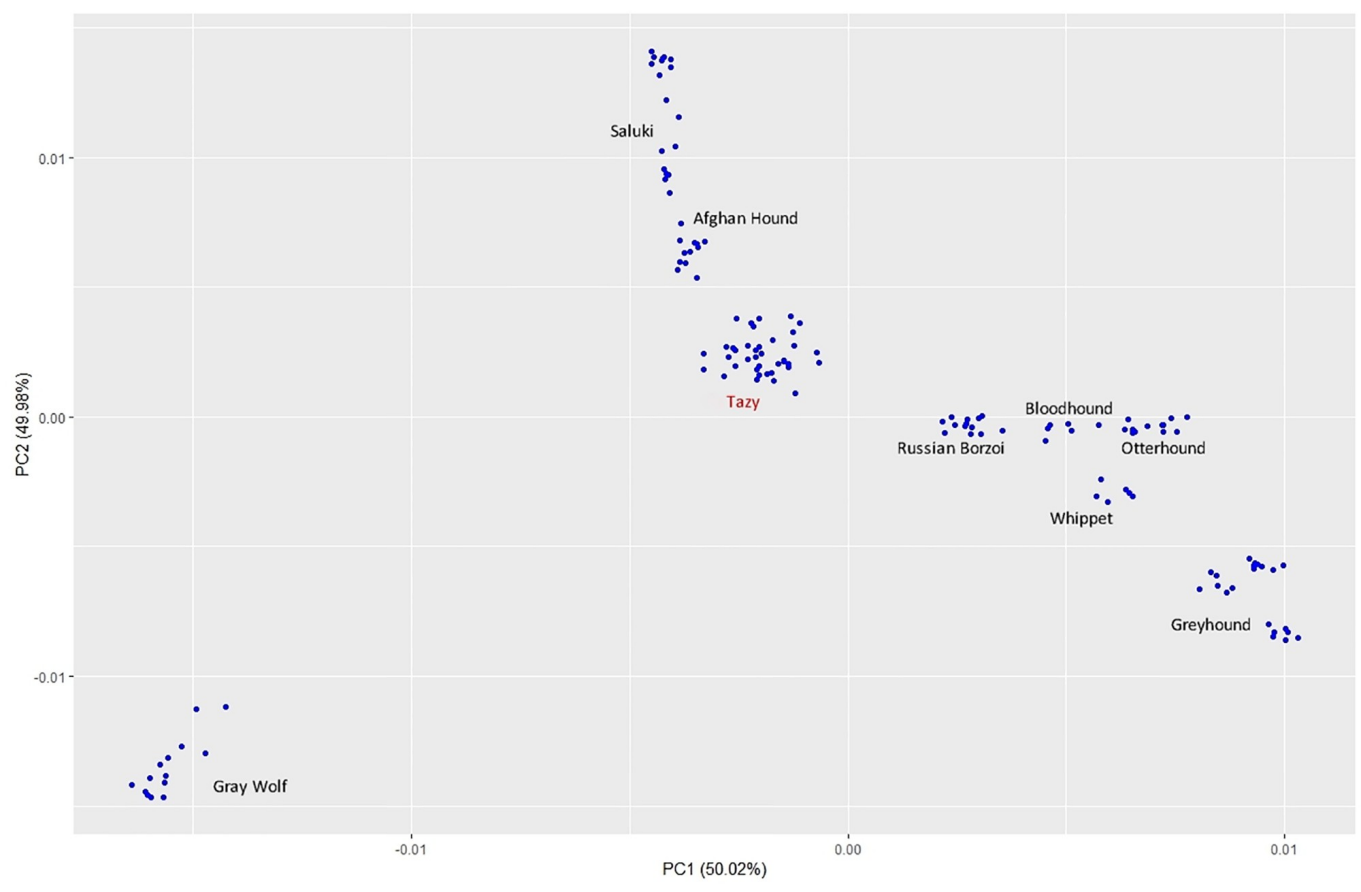
Dog clustered based on PCoA using individual SNP genotypes. Plot for the first (PC1) and second (PC2) component revealed the clustering of 39 Tazy dogs, 74 dogs from seven sighthound breeds and 14 Grey Wolves.

Since Bayesian clustering of the STR dataset did not reveal any divergence between the Tazy and the Saluki, we performed SNP-based analyses for 39 Tazy dogs. The resulting dataset was merged with publicly available SNP data for 89 dogs from seven sighthound breeds and 14 Grey Wolves. Relationships between breeds were visualized using PCoA and the phylogenetic dendrogram. The PCoA plot shows stratification between breeds and no significant hybridization of sighthounds with wolves (Fig 5). The eight sighthounds formed eight groups. There was a clear genetic split between Tazy and Saluki. The Afghan Hound was found between the Tazy and the Saluki, confirming the results of the tree plot and PCoA based on the data from STR. The Russian Borzoi, the Bloodhound, the Otterhound, the Whippet and the Greyhound were closer and had a distance to the Tazy, the Afghan Hound and the Saluki.

**Fig 6:**
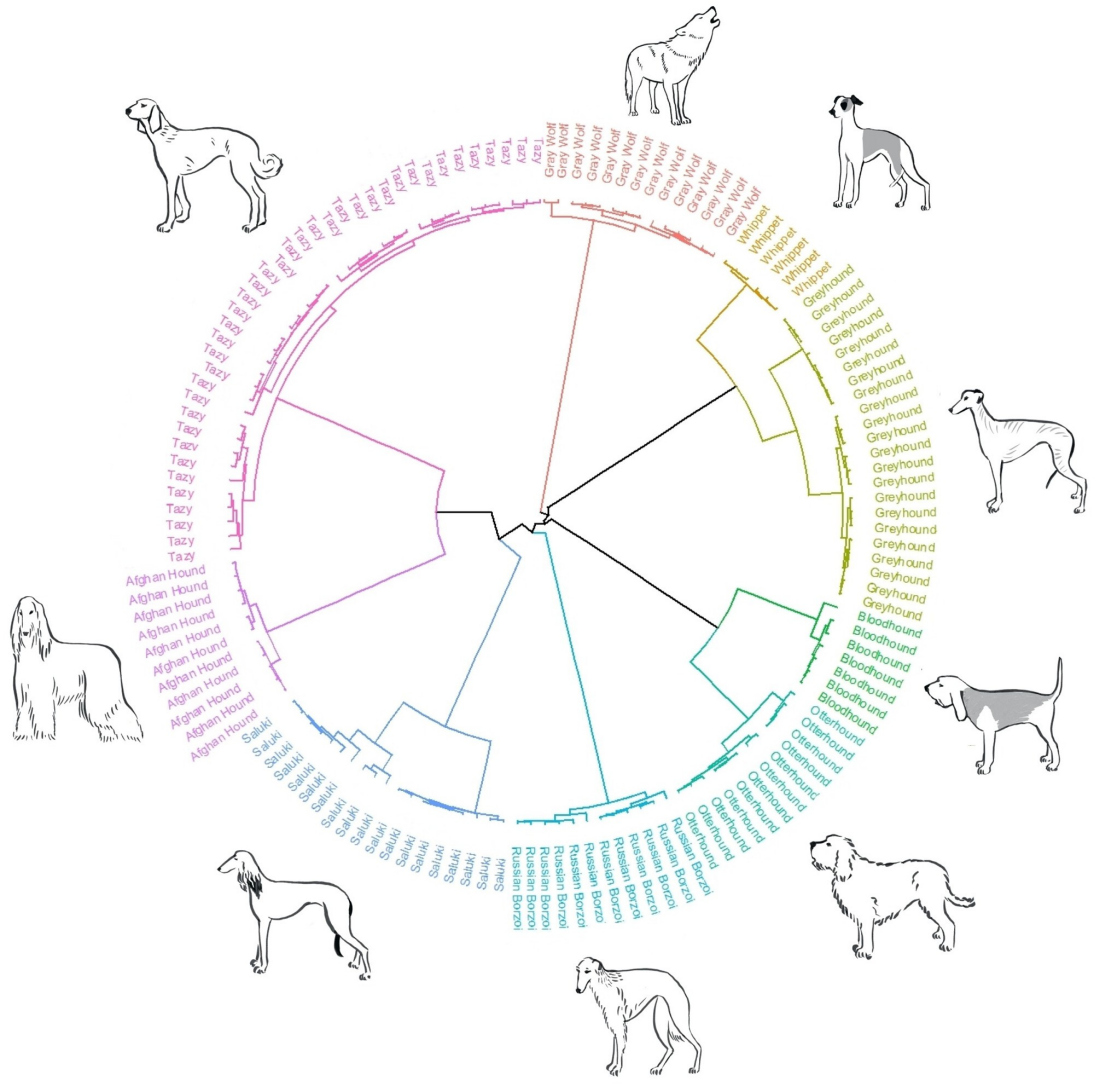
Phylogenetic dendrogram based on SNP markers for breed relationship definition. Phylogenetic network constructed using complete linkage hieratical clustering based on 40,229 autosomal SNPs for 39 Tazy dogs, 89 dogs from seven sighthound breeds and 14 Grey Wolves. Breeding lines labels were color-coded. Canine images not drawn to scale. Reprinted from https://drive.google.com/drive/folders/1hFzoHRWnQ7-eNeKDZKwBy_nLaUs3bTJY?usp=share_link] under a CC BY license, with permission from Anna Molodykh, original copyright 2022.

In the phylogenetic tree, the Tazy comes from the same branchpoint with the Afghan Hound and the Saluki (Fig 6), showing that there is an ancient common ancestry between these three breeds, but a divergence between the Tazy/Afghan Hound and the Saluki, and then a more recent divergence between the Tazy and the Afghan Hound. Similarly, the Greyhounds and the Whippets share a common ancestral gene pool, as well as the Otterhound and the Bloodhound.

## Discussion

This is the first study describing the genetic structure of the Tazy breed. We focused on three regions of Kazakhstan, where the Tazy has been an important hunting dog for many centuries, to evaluate the parameters of genetic diversity. Using the 18 STR-marker panel, more than 100 Tazy dogs were genotyped. The results show that the breed has a high level of genetic diversity. The number of alleles that have the greatest genetic influence on heterozygosity, known as Ne, averaged 4.869 in the general population, which is higher than the Ne reported for other native dog breeds: 3.0–3.5 for the Italian Greyhound [28], 3.3 for the German Shepherd, 3.5 for the Maltese, 3.2 for the Biewer Yorkshire Terrier, 3.5 for the Yorkshire Terrier [29]. All markers were highly informative (PIC values greater than 0.5) [30], suggesting the efficacy of these markers for the assessment of genetic variation among the Tazy dogs. The average polymorphism value based on all Tazy dogs was 0.739, a value very similar to that obtained for Greyhounds from South Korea (0.73) [13]. In contrast, the PIC value in a Polish Greyhound population was much lower (0.597), as reported by Goleman et al. [31]. The highest level of polymorphism was observed at the REN169D01 and AHT121 loci, where the PIC value exceeded 0.850. An equally high degree of polymorphism of these markers was found in 28 Borzoi dogs in Poland (PIC = 0.756 and 0.732, respectively) [32]. The lowest level of polymorphism was observed for the marker REN247M23, with a PIC value of 0.543. A study of 28 borzoi dogs also revealed one of the lowest levels of polymorphisms of this marker, with a PIC value of 0.590 [32]. We also found the lowest heterozygosity for this marker (Ho = 0.518). The mean observed heterozygosity calculated for all STR was 0.748. A similar degree of heterozygosity was found for Jack Russell Terriers (Ho = 0.75), Yorkshire Terriers (Ho = 0.73), and mixed breeds (Ho = 0.73) [33]. A study of Italian Greyhounds found lower Ho values in the range of 0.60–0.62 [28]. The estimated He values were slightly higher than the Ho values for the whole population and for the Tazy of the Southern and Northern region. Thus, the fixation index values were positive, indicating inbreeding, while the mean F value for the Tazy of the Eastern region had a negative value (-0.121), indicating that the dogs were not inbred. However, the detected inbreeding was low and did not exceed the value considered extremely high (> 0.1) [34].

The obtained high degree of diversity and allelic richness suggests that the Tazy breed evolved from a relatively large and diverse founder population. Moreover, the intra-breed substructure of the Tazy identified in the STRUCTURE bar plot may be the result of different ancestors. The high diversity inherited from the ancestors was maintained despite possible genetic bottlenecks in the last century, when the Tazy lost importance for hunting, and may be explained by gene flows between populations. The STRUCTURE bar plot clearly shows migration events and exchange of genetic material between all three regions, which can be explained by the historically evolved lifestyle of the Kazakhs. The semi-nomadic way of life with seasonal migrations of people with their dogs favored a dynamic gene flow, which was partially restricted by landscape barriers. However, despite high genetic diversity, the Tazy showed low differentiation when the Tazy dogs were compared with purebred sighthounds (Fst = 0.0033), indicating weak effects of reproductive isolation. Similarly, low genetic differentiation and high diversity were also found for Portuguese native breeds and were interpreted by the fact that these breeds are not closed populations [35].

Of particular interest was the comparison between the Tazy and the phenotypically similar Saluki. Bayesian clustering on a microsatellite data set showed that by the third cluster calculation, the Tazy breed already differs from the Greyhound, Afghan Hound, Whippet, and Russian Borzoi, but has an identical profile to the Saluki. Parker concludes that breeds that do not form distinct clusters by microsatellite analysis, but tend to cluster with another breed, have only recently been separated [9]. We then used the HD SNP array to create a high-resolution population structure of the Tazy and compared the obtained data in a broader context of worldwide sighthound breeds. The Tazy appeared to be genetically distant from all other breeds included in our study and was part of the eastern sighthound group along with the Saluki and Afghan Hound. There was a clear separation between the Tazy and the Saluki dogs. In the phylogenetic dendrogram, the Tazy, the Afghan Hound and the Saluki breeds shared one clade, and the Tazy formed a subclade with the Afghan Hound. The close relationship of the Tazy to the Afghan Hound was to be expected given the phenotypic similarity. A local name for the Afghan Hound, Sag-e-Tazi, suggests a possible common ancestry with the Tazy. Interestingly, in the phylogenetic dendrogram, only the Saluki was previously closely related to the Afghan Hound [8, 9, 17]. These two breeds are considered to be an "ancient" breed based on several genetic studies [9, 36] and are believed to have originated > 500 years ago [17, 37]. Apparently, the Tazy is the missing link in this group of ancient breeds. The hypothesis that the Tazy is a dog with a centuries-old history is consistent with archaeological findings. Numerous images of Tazy-like dogs on petroglyphs in South Kazakhstan date from different historical periods, up to the 10th-12th centuries [5].

Moreover, the results of other significant branching were in clear agreement with the known history of the designated breed. In our dendrogram, the Otterhound and the Bloodhound share a common ancestral gene pool. The Otterhound is an ancient breed that evolved in England from Bloodhounds or other southern hunting dogs, which could be the common ancestor of these two breeds [38]. Other breeds that were grouped together are the Greyhound and the Whippet, which is consistent with the Parker results [9]. It is believed that the breed evolved from terriers and small English Greyhounds and was later crossed with Italian Greyhounds to give the Whippet a sleek appearance [39].

The strong limitation of the study is that the number of samples obtained from sighthound breeds for the STR analysis was very small due to the lower popularity of these breeds in our country. We are aware that a small number of samples should not be used to achieve adequate power in admixture mapping studies. With a smaller sample size, a study may not detect small or moderate effects [40]. However, in this study, the STR analysis was able to distinguish the Greyhound, Afghan Hound, Whippet, and Russian Borzoi with a small number of samples. This is evidence that genetic clustering algorithms implemented in STRUCTURE can be successfully used to characterize individuals and populations previously separated with minimal genetic contact over hundreds of years. A few studies support the successful clustering of breeds based on only a few representatives of each breed [9, 41, 42]. In addition, we present STRUCTURE and PCoA results based on STR markers alongside PCoA results based on datasets of 170,000 marker genotyping arrays to test whether the assumptions of the model are likely to hold and to validate certain features of the results. We hope that this study is the beginning of a detailed demographic and historical analysis of the Tazy dog breed. Future studies on the Tazy should focus on the comprehensive study of mtDNA, genetic selection of hunting success and determination of disease genes.

## Conclusion

The Tazy breed consists of individuals with specific morphological and behavioural characteristics and represents an undeniable genetic and cultural heritage. The history of the Tazy and their role as hunters are well documented. In this study, we used data from STR and SNP sources to study the genome structure of the Tazy breed. We characterized the Tazy as a unique breed with a specific population structure. Genetic analysis shows that the Tazy have high genetic diversity. One of the most important results of our genetic study is the identification of the Tazy as a genetically divergent ancient dog breed with a strong position in the phylogenetic tree. These results provide the first scientific basis for genetic data to improve and maintain this breed in the future and support the proposal for international recognition of the Tazy.

## Supporting information

S1 Fig Pictures of studied Tazy. (PDF)

S1 Table Breed standard of the Tazy breed (the Republican Federation of Public Associations of Hunters and Hunting Societies "Kansonar" and the Ministry of Agriculture of the Republic of Kazakhstan). (PDF)

S2 Table SentrixBarcode and SentrixPosition of the Tazy samples on the Illumina Infinium CanineHD Genotyping BeadChip. (PDF)

S3 Table The sample code and corresponding breed (datadryad.org). (PDF)

S4 Table Tazy dogs and cluster assignment. (PDF)

S5 Table Sighthound dogs and cluster assignment. (PDF)
