# Supplementary material for: Kazakh national dog breed Tazy: What do we know?
Source: PLoS One. 2023 Mar 8;18(3):e0282041. doi: 10.1371/journal.pone.0282041 (PMC9994743; doi:10.1371/journal.pone.0282041)
Supplement: S1 Fig — (PDF) [file pone.0282041.s001.pdf]

Kazakh National dog breed Tazy: what do we know?

Anastassiya Perfilyeva[1], Kira Bespalova[1,2], Sergey Bespalov[3], Mamura Begmanova[1], Yelena Kuzovleva[1], Zhassulan Zhaniyazov[1,2], Olga Vishnyakova[4], Inna Nazarenko[5], Yuliya Perfilyeva[6], Ozada Khamdiyeva[1], Bakhytzhan Bekmanov[1,2]

[1] Department of Molecular Genetics, Institute of Genetics and Physiology, Almaty, Kazakhstan

[2] Department of Biology and Biotechnology, Al-Farabi Kazakh National University, Almaty, Kazakhstan

[3] Department of Theriology, Institute of Zoology, Almaty, Kazakhstan

[4] Department of Cynology, Republican Federation of Public Associations of Hunters and Hunting Societies "Kansonar", Almaty, Kazakhstan

[5] Department of Cynology, Republican Federation of Public Associations of Hunters and Hunting Societies "Kansonar", Astana, Kazakhstan

[6] Department of Immunology, M.A. Aitkhozhin's Institute of Molecular Biology and Biochemistry, Almaty, Kazakhstan

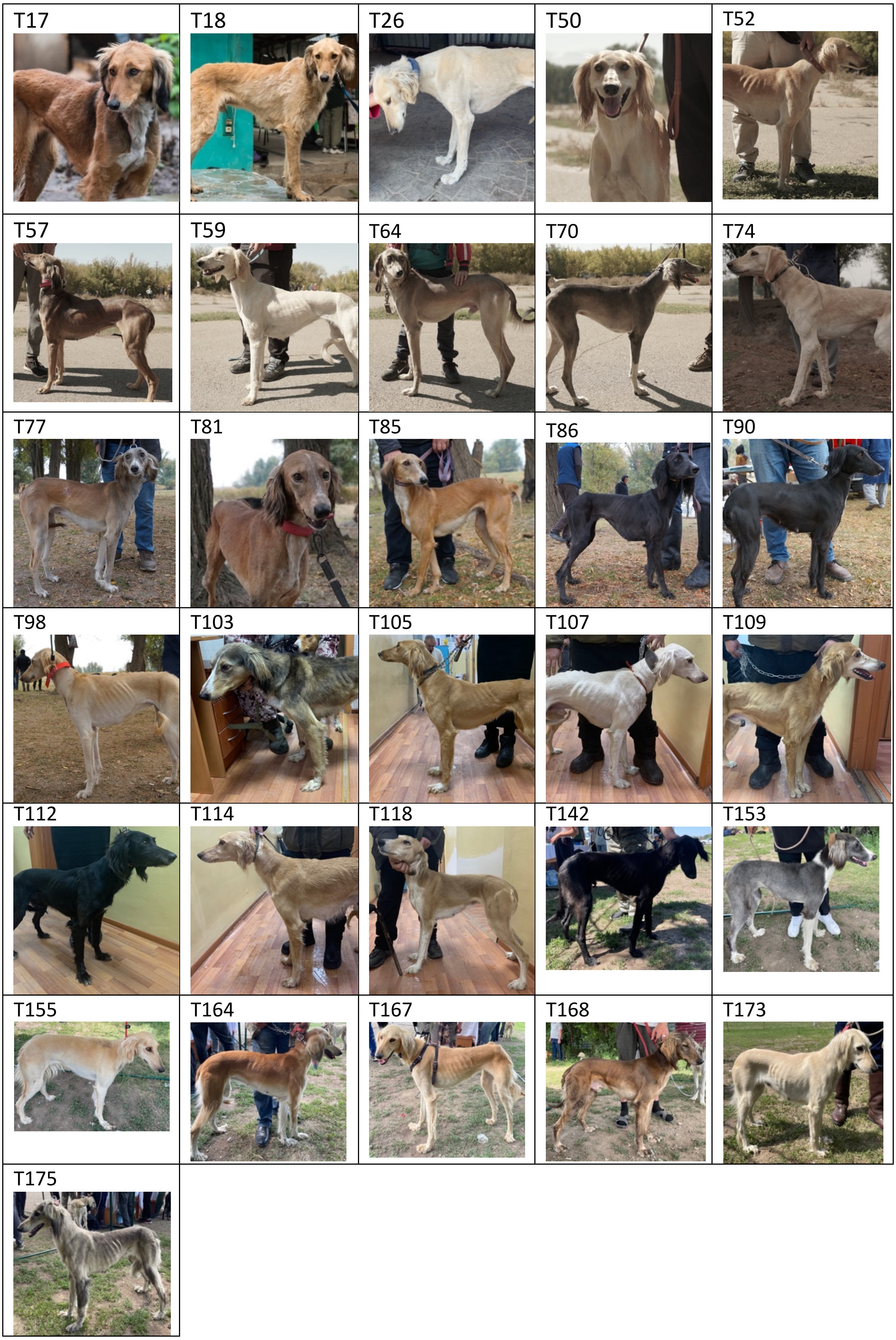

S1 Fig. Pictures of studied Tazy
