# Supplementary material for: Kazakh national dog breed Tazy: What do we know?
Source: PLoS One. 2023 Mar 8;18(3):e0282041. doi: 10.1371/journal.pone.0282041 (PMC9994743; doi:10.1371/journal.pone.0282041)
Supplement: S1 Table — (PDF) [file pone.0282041.s002.pdf]

S1 Table. Breed standard of the Tazy breed (the Republican Federation of Public Associations of Hunters and Hunting Societies "Kansonar" and the Ministry of Agriculture of the Republic of Kazakhstan).

N		Standard	Disadvantages	Faults
1.	General appearance	Medium and above average size, dry, strong type, the length/height ratio is 103. The height at the withers: 60-70 cm for dogs and 55-65 cm for bitches.	Excessively delicate constitution, high on leg, the small length/height ratio (up to 106)	Loose or coarse constitution, the length/height ratio less than 100 or more than 106.
2.	Size	Exceeding the upper growth limit is not a disadvantage as long as the proportions and other requirements of the standard are met.	Height at withers up to 2 centimeters below the height specified in the standard.	The back is above the withers
3.	Colour	Black, white, straw color in various shades, red and gray in all shades. Possible black spots on muzzle, ears and lower part of legs. Puppies may change color during growth and puberty.	Piebald color, red tan	Brindle, mottled or merle colour, auburn and reddish tan
4.	Coat	The coat is soft, silky, straight, short, up to 4.5 cm long (in winter) and up to 2.5 cm (in summer) with well developed undercoat. On the ears there are soft, wavy, elongated hairs that hang down 5-6 cm or more below the tips, forming the so-called "burkas". The elongated hairs on the back of the forelegs and thighs form feathering. On the underside of the tail there is a sparse dewlap (up to 10 cm). Short and wire hairs grow between the fingers. Smooth-haired tazy is allowed (without burka and suspension). If they meet the other parameters of the standard, smooth coat is neither a defect nor a disadvantage.	Absence of burkas or feathering, long hair all over the body (more than 5 cm), partial long hair on the sides, on the sides of the hind legs.	Curly, broken, wavy coat, tufts on head, long hair on belly and chest, thick dewlap on right side.

5.	Skin, muscles, bones	The skin is thin, elastic, without wrinkles, the muscles are well developed, especially on the hips and loin, the bones are substantial but not heavy	Weak or slightly coarse bone. Underdeveloped muscles	Bone too coarse. Thick skin that forms a dewlap or wrinkles. Dogs of the bitch type. Bitches of the dog type.
6.	Head and skull	Dry, elongated, with a moderately wide skull. Seen from above, the head is wedge-shaped. The occiput is moderately developed, the parietal crest is weakly developed. The transition from the forehead to the muzzle (stop) is smooth. The muzzle is dry, equal or slightly shorter than the length of the skull, slightly pointed with a small hump or straight, without socket or furrows, well filled under the eyes. The cranial part is flattened from above. The line of the muzzle is parallel to the line of the skull or slightly lowered. The lips are thin and tight. Jaws are straight, well developed with large, healthy teeth.	Short head, excessively developed brows and cheeks, clear transition from forehead to muzzle (stop), no tight lips	Coarse, wet, cheeky head, bulging skull, broken up face, muzzle longer than the skull. The line of the head is curved upwards. Raw, pendulous lips. Overshot or undershot bite.
7.	Ears	Dropped, thin, raised at cartilage, set at or slightly above the outer corner of the eye, covered with soft, wavy hair. The hair hangs down 5-6 centimeters below the tips of the ears. The tips of the ears reach the corners of the lips. When excited, tips lift slightly at the cartilage, causing the ear to move to the side.	Ears shortened (up to 2 centimeters not reaching the corners of the lips), excessively raised at the cartilage.	Short (not reaching the corner of the lips by more than 2 centimeters), set too high or too low, semi-dropped, rose, thick, dead
8.	Eyes	large, slightly slanting, brown, dark brown, sometimes prominent, the cut is almond-shaped, the eyelids are black	not large; sunken or bulging; bright; the edges of the eyelids are pale	small, sunken, straight-set, discordant, yellow or gray in color; pink eyelids.
9.	Nose	Black, brown is allowed if light colour	Poorly pigmented	Pink

10.	Teeth	Large, white, scissors bite, level bite is allowed after 6 years	Missing first premolar (P1) in the mandible	The incisors are not in line, the absence of any tooth, except for the lower first premolar (P1).
11.	Neck	Round or slightly compressed laterally, medium or long, sometimes slightly upsweep (swan neck). Medium, strong is preferable. Upright carriage of the neck	Wet, short	Excessively short, with dewlap
12.	Chest	Oval, wide, deep; seen from the front, the body fits between the hind legs; its underside near the front legs reaches the horizontal line of the elbow joints; the chest is distinctly narrowed toward the abdomen	Too narrow, not wider than the croup; not deep enough, not more than 3 inches to the elbows; wide open	Flat ribs; pronounced keel bone; short chest not projecting beyond elbows; shallow brisket, not reaching the elbows by more than 3 centimeters
13.	Withers	Rises slightly above the backline and forms a socket behind the scapulas	Easily visible withers	No visible withers
14.	Back	The back is wide, elastic, muscular, almost straight. Together with the loin, it forms a slight projection upwards.	Narrow, soft, weak muscles	Hollow or humped back
15.	Loin	Short, arched, with relief muscles.	Straight, weak, narrow, long, with insufficiently developed muscles.	Narrow, excessively arched, with underdeveloped muscles
16.	Croup	Broad, long, slightly sloping; the musculature is sculpted and strong, the width between the huckles is at least 7 cm.	Short, narrow, overly sloping	Horizontal
17.	Abdomen	Tucked-up	No tucked-up	Paunchy
18.	Forequarters	Long, straight and parallel; scapula articulates with the humerus with an angle of 90-100°; the pasterns are relatively long and slightly oblique.	Out at or out in elbows, light east-west feet or toeing-in. Short, weak, too oblique pasterns. The scapula forms an angle of more than 110° with the humerus.	Round bones in cross section, curved limbs, straight in shoulders, elbows turned strongly outward or inward, east-west feet or toeing-in. Up-right pastern or knuckling

19.	Hindquarters	The hind feet are dry, bony, with long levers, well angulated and straight and parallel when viewed from behind. The hocks are well developed, set wide apart when standing, so that the metatarsals are almost vertical. The metatarsals are long. Strongly developed muscles on the thighs	Lack angulated; wide or narrow rear views. Short metatarsals. Underdeveloped muscles. Slight convergence or unfolding of the hocks.	Poorly angulated, musculature underdeveloped, hocks close together, paws turned outward (cow hocked or pigeon-toed, bowed rear views). Rear dewclaws.
20.	Feet	Strong, oval. Closely knit toes, claws down to the ground.	Slight split-up toes, claws not in the ground	Cat foot, flat feet, well split-up toes
21.	Tail	The tail is thin, sabre-shaped, unfolded to the hock or slightly shorter, bent at the end into a steep hook or folded into a small ring. Raised slightly above the backline when moving	Long or short; thick; rolled on its side; sickle-shaped, with a small ring or hook at the end	Turned over the back tail, fused coccygetal vertebrae at the end, with folds, no ring or hook at the end, vertical tail post
22.	Gait/movement	Gentle trot, the fastest gait when chasing an animal		Pace
23.	Temperament	Agility, vigilance, viciousness to the beast, endurance and maneuverability		

24. Disqualifying faults: aggressiveness toward humans; cowardice; malocclusions; absence of incisor(s), canine(s), premolar(s) (except lower first premolar), and maxillary and/or mandibular molar(s) (in the absence of trauma); different colored eyes; blue eyes; coffee color; dewclaws on hind legs; cryptorchidism; dysplasia.
