# Supplementary material for: Kazakh national dog breed Tazy: What do we know?
Source: PLoS One. 2023 Mar 8;18(3):e0282041. doi: 10.1371/journal.pone.0282041 (PMC9994743; doi:10.1371/journal.pone.0282041)
Supplement: S2 Table — (PDF) [file pone.0282041.s003.pdf]

S2 Table. SentrixBarcode and SentrixPosition of the Tazy samples on the Illumina Infinium CanineHD Genotyping BeadChip.

Code	SentrixBarcode_A	SentrixPosition_A
T17	206339310006	R01C01
T18	206339310006	R02C01
T26	206339310006	R03C01
T50	206339310006	R04C01
T52	206339310006	R05C01
T57	206339310006	R06C01
T74	206339310006	R01C02
T59	206339310006	R02C02
T77	206339310006	R03C02
T98	206339310006	R04C02
T103	206339310006	R05C02
T105	206339310006	R06C02
T107	206420890009	R01C01
T114	206420890009	R02C01
T118	206420890009	R03C01
T130	206420890009	R04C01
T142	206420890009	R01C02
T164	206420890009	R02C02
T173	206420890009	R03C02
T175	206420890009	R04C02
T176	206420890009	R05C02
T177	206420890009	R06C02
T64	206339310005	R01C01
T70	206339310005	R02C01
T81	206339310005	R03C01
T84	206339310005	R04C01
T85	206339310005	R05C01
T86	206339310005	R06C01
T90	206339310005	R01C02
T109	206339310005	R02C02
T112	206339310005	R03C02
T120	206339310005	R04C02
T138	206339310005	R05C02
T150	206339310005	R06C02
T153	206339310004	R01C01
T155	206339310004	R02C01
T167	206339310004	R03C01
T168	206339310004	R04C01
T178	206339310004	R06C01
