# Supplementary material for: Kazakh national dog breed Tazy: What do we know?
Source: PLoS One. 2023 Mar 8;18(3):e0282041. doi: 10.1371/journal.pone.0282041 (PMC9994743; doi:10.1371/journal.pone.0282041)
Supplement: S3 Table — (PDF) [file pone.0282041.s004.pdf]

S3 Table. The sample code and corresponding breed (datadryad.org).

N	Breed	Code	Link
1	Afghan Hound	PFZ1E05	doi_10.5061_dryad.v9t5h
2	Afghan Hound	PFZ5A11	doi_10.5061_dryad.v9t5h
3	Afghan Hound	PFZ5B08	doi_10.5061_dryad.v9t5h
4	Afghan Hound	PFZ5B11	doi_10.5061_dryad.v9t5h
5	Afghan Hound	PFZ5C08	doi_10.5061_dryad.v9t5h
6	Afghan Hound	PFZ5G04	doi_10.5061_dryad.v9t5h
7	Afghan Hound	PFZ16A02	doi_10.5061_dryad.v9t5h
8	Afghan Hound	PFZ16D01	doi_10.5061_dryad.v9t5h
9	Afghan Hound	PFZ16G05	doi_10.5061_dryad.v9t5h
10	Afghan Hound	PFZ17B12	doi_10.5061_dryad.v9t5h
11	Afghan Hound	PFZ17F09	doi_10.5061_dryad.v9t5h
12	Bloodhound	PFZ10C12	doi_10.5061_dryad.v9t5h
13	Bloodhound	PFZ11A11	doi_10.5061_dryad.v9t5h
14	Bloodhound	PFZ11C05	doi_10.5061_dryad.v9t5h
15	Bloodhound	PFZ11D09	doi_10.5061_dryad.v9t5h
16	Bloodhound	PFZ11D12	doi_10.5061_dryad.v9t5h
17	Bloodhound	PFZ11H12	doi_10.5061_dryad.v9t5h
18	Bloodhound	PFZ42E04	doi_10.5061_dryad.v9t5h
19	Gray Wolf	UCLA_Cr6	doi_10.5061_dryad.v9t5h
20	Gray Wolf	UCLA_In3	doi_10.5061_dryad.v9t5h
21	Gray Wolf	UCLA_Isr	doi_10.5061_dryad.v9t5h
22	Gray Wolf	Wlf_19785	doi_10.5061_dryad.v9t5h
23	Gray Wolf	Wlf_22799	doi_10.5061_dryad.v9t5h
24	Gray Wolf	Wlf_22800	doi_10.5061_dryad.v9t5h
25	Gray Wolf	Wlf_22802	doi_10.5061_dryad.v9t5h
26	Gray Wolf	Wlf_22803	doi_10.5061_dryad.v9t5h
27	Gray Wolf	Wlf_22809	doi_10.5061_dryad.v9t5h
28	Gray Wolf	Wlf_22810	doi_10.5061_dryad.v9t5h
29	Gray Wolf	Wlf_LU1656	doi_10.5061_dryad.v9t5h
30	Gray Wolf	Wlf_LU1657	doi_10.5061_dryad.v9t5h
31	Gray Wolf	Wlf_LUb1	doi_10.5061_dryad.v9t5h
32	Gray Wolf	Wlf_LUb3	doi_10.5061_dryad.v9t5h
33	Greyhound	Gry_GT238	doi_10.5061_dryad.v9t5h
34	Greyhound	Gry_GT239	doi_10.5061_dryad.v9t5h
35	Greyhound	Gry_GT240	doi_10.5061_dryad.v9t5h
36	Greyhound	Gry_GT242	doi_10.5061_dryad.v9t5h
37	Greyhound	Gry_GT243	doi_10.5061_dryad.v9t5h
38	Greyhound	Gry_GT244	doi_10.5061_dryad.v9t5h
39	Greyhound	Gry_GT245	doi_10.5061_dryad.v9t5h
40	Greyhound	Gry_GT246	doi_10.5061_dryad.v9t5h
41	Greyhound	Gry_GT247	doi_10.5061_dryad.v9t5h
42	Greyhound	Gry_GT248	doi_10.5061_dryad.v9t5h
43	Greyhound	Gry_GT249	doi_10.5061_dryad.v9t5h
44	Greyhound	PFZ23B05	doi_10.5061_dryad.v9t5h
45	Greyhound	PFZ24D05	doi_10.5061_dryad.v9t5h
46	Greyhound	PFZ24E06	doi_10.5061_dryad.v9t5h
47	Greyhound	PFZ24G06	doi_10.5061_dryad.v9t5h

48	Greyhound	PFZ26B06	doi_10.5061_dryad.v9t5h
49	Greyhound	PFZ26C06	doi_10.5061_dryad.v9t5h
50	Greyhound	PFZ26D05	doi_10.5061_dryad.v9t5h
51	Greyhound	PFZ26E05	doi_10.5061_dryad.v9t5h
52	Greyhound	PFZ42A08	doi_10.5061_dryad.v9t5h
53	Greyhound	PFZ42F07	doi_10.5061_dryad.v9t5h
54	Greyhound	PFZ43F01	doi_10.5061_dryad.v9t5h
55	Otterhound	PFZ16B07	doi_10.5061_dryad.v9t5h
56	Otterhound	PFZ16C07	doi_10.5061_dryad.v9t5h
57	Otterhound	PFZ17C03	doi_10.5061_dryad.v9t5h
58	Otterhound	PFZ17H04	doi_10.5061_dryad.v9t5h
59	Otterhound	PFZ36A05	doi_10.5061_dryad.v9t5h
60	Otterhound	PFZ36B05	doi_10.5061_dryad.v9t5h
61	Otterhound	PFZ36D04	doi_10.5061_dryad.v9t5h
62	Otterhound	PFZ36E04	doi_10.5061_dryad.v9t5h
63	Otterhound	PFZ36F04	doi_10.5061_dryad.v9t5h
64	Otterhound	PFZ36G04	doi_10.5061_dryad.v9t5h
65	Otterhound	PFZ36H04	doi_10.5061_dryad.v9t5h
66	Otterhound	PFZ17D03	doi_10.5061_dryad.v9t5h
67	Russian Borzoi	PFZ4C03	doi_10.5061_dryad.v9t5h
68	Russian Borzoi	PFZ4D03	doi_10.5061_dryad.v9t5h
69	Russian Borzoi	PFZ4E03	doi_10.5061_dryad.v9t5h
70	Russian Borzoi	PFZ4F03	doi_10.5061_dryad.v9t5h
71	Russian Borzoi	PFZ4G05	doi_10.5061_dryad.v9t5h
72	Russian Borzoi	PFZ4H05	doi_10.5061_dryad.v9t5h
73	Russian Borzoi	PFZ5D08	doi_10.5061_dryad.v9t5h
74	Russian Borzoi	PFZ5E10	doi_10.5061_dryad.v9t5h
75	Russian Borzoi	PFZ5F10	doi_10.5061_dryad.v9t5h
76	Russian Borzoi	PFZ5H01	doi_10.5061_dryad.v9t5h
77	Russian Borzoi	PFZ19A03	doi_10.5061_dryad.v9t5h
78	Russian Borzoi	PFZ20H01	doi_10.5061_dryad.v9t5h
79	Saluki	PFZ6D04	doi_10.5061_dryad.v9t5h
80	Saluki	PFZ6F08	doi_10.5061_dryad.v9t5h
81	Saluki	PFZ6G08	doi_10.5061_dryad.v9t5h
82	Saluki	PFZ7E12	doi_10.5061_dryad.v9t5h
83	Saluki	PFZ7F12	doi_10.5061_dryad.v9t5h
84	Saluki	PFZ42A07	doi_10.5061_dryad.v9t5h
85	Saluki	PFZ42A09	doi_10.5061_dryad.v9t5h
86	Saluki	SAL-1	doi_10.5061_dryad.pm7mt
87	Saluki	SAL-2	doi_10.5061_dryad.pm7mt
88	Saluki	SAL-3	doi_10.5061_dryad.pm7mt
89	Saluki	SAL-4	doi_10.5061_dryad.pm7mt
90	Saluki	SAL-5	doi_10.5061_dryad.pm7mt
91	Saluki	SAL-6	doi_10.5061_dryad.pm7mt
92	Saluki	SAL-7	doi_10.5061_dryad.pm7mt
93	Saluki	SAL-8	doi_10.5061_dryad.pm7mt
94	Saluki	SAL-9	doi_10.5061_dryad.pm7mt
95	Saluki	SAL-10	doi_10.5061_dryad.pm7mt
96	Saluki	SAL-11	doi_10.5061_dryad.pm7mt
97	Saluki	SAL-12	doi_10.5061_dryad.pm7mt
98	Whippet	PFZ14E03	doi_10.5061_dryad.v9t5h
99	Whippet	PFZ14G11	doi_10.5061_dryad.v9t5h
100	Whippet	PFZ14H09	doi_10.5061_dryad.v9t5h

101	Whippet	PFZ22G05	doi_10.5061_dryad.v9t5h
102	Whippet	PFZ42B09	doi_10.5061_dryad.v9t5h
103	Whippet	PFZ42E06	doi_10.5061_dryad.v9t5h
