# Supplementary material for: Kazakh national dog breed Tazy: What do we know?
Source: PLoS One. 2023 Mar 8;18(3):e0282041. doi: 10.1371/journal.pone.0282041 (PMC9994743; doi:10.1371/journal.pone.0282041)
Supplement: S4 Table — (PDF) [file pone.0282041.s005.pdf]

S4 Table. Tazy dogs and cluster assignment.

N	Code	Cluster1	Cluster2	Cluster3
1	T1	0.662	0.156	0.181
2	T2	0.221	0.467	0.312
3	T6	0.083	0.775	0.141
4	T7	0.085	0.848	0.066
5	T10	0.073	0.505	0.422
6	T11	0.846	0.079	0.075
7	T12	0.719	0.087	0.194
8	T13	0.566	0.260	0.174
9	T15	0.825	0.058	0.117
10	T17	0.534	0.239	0.227
11	T18	0.250	0.552	0.197
12	T19	0.630	0.237	0.133
13	T21	0.063	0.655	0.282
14	T22	0.429	0.207	0.364
15	T23	0.771	0.089	0.140
16	T25	0.093	0.853	0.055
17	T26	0.442	0.080	0.479
18	T27	0.400	0.310	0.289
19	T28	0.318	0.154	0.528
20	T29	0.127	0.490	0.382
21	T30	0.626	0.070	0.303
22	T33	0.196	0.407	0.397
23	T36	0.217	0.587	0.196
24	T43	0.234	0.500	0.266
25	T44	0.621	0.194	0.185
26	T45	0.314	0.334	0.352
27	T46	0.496	0.125	0.380
28	T47	0.275	0.326	0.399
29	T48	0.312	0.067	0.621
30	T49	0.344	0.147	0.508
31	T50	0.188	0.083	0.729
32	T51	0.745	0.091	0.164
33	T52	0.327	0.178	0.495
34	T53	0.293	0.480	0.227
35	T54	0.277	0.193	0.530
36	T55	0.166	0.452	0.382
37	T56	0.187	0.455	0.359
38	T57	0.766	0.080	0.155
39	T58	0.167	0.644	0.188
40	T59	0.280	0.306	0.414
41	T60	0.137	0.285	0.579
42	T61	0.332	0.095	0.573
43	T62	0.409	0.062	0.529
44	T63	0.254	0.442	0.304
45	T64	0.189	0.084	0.727
46	T65	0.186	0.056	0.759
47	T66	0.270	0.583	0.147
48	T67	0.526	0.220	0.254

49	T68	0.128	0.416	0.456
50	T69	0.254	0.143	0.604
51	T70	0.160	0.160	0.680
52	T71	0.221	0.272	0.507
53	T72	0.227	0.149	0.624
54	T73	0.477	0.401	0.122
55	T136	0.674	0.104	0.222
56	T137	0.853	0.082	0.064
57	T179	0.099	0.082	0.819
58	T182	0.038	0.105	0.858
59	T74	0.092	0.629	0.279
60	T75	0.130	0.715	0.154
61	T76	0.324	0.564	0.112
62	T78	0.102	0.715	0.183
63	T79	0.085	0.622	0.293
64	T80	0.184	0.405	0.411
65	T81	0.128	0.454	0.419
66	T82	0.102	0.784	0.114
67	T83	0.269	0.570	0.161
68	T84	0.459	0.461	0.079
69	T85	0.141	0.426	0.433
70	T86	0.178	0.756	0.066
71	T87	0.152	0.323	0.525
72	T88	0.113	0.182	0.705
73	T89	0.131	0.780	0.088
74	T90	0.081	0.539	0.380
75	T91	0.223	0.454	0.323
76	T92	0.076	0.792	0.132
77	T93	0.140	0.476	0.384
78	T94	0.252	0.452	0.296
79	T95	0.252	0.472	0.276
80	T96	0.201	0.677	0.122
81	T97	0.225	0.364	0.411
82	T98	0.150	0.475	0.375
83	T99	0.519	0.399	0.082
84	T100	0.055	0.879	0.066
85	T101	0.570	0.361	0.070
86	T102	0.093	0.293	0.614
87	T103	0.073	0.104	0.823
88	T105	0.056	0.055	0.888
89	T107	0.787	0.080	0.134
90	T108	0.335	0.483	0.183
91	T109	0.750	0.094	0.156
92	T110	0.235	0.666	0.099
93	T111	0.154	0.267	0.579
94	T112	0.075	0.253	0.673
95	T113	0.076	0.144	0.780
96	T114	0.190	0.153	0.656
97	T116	0.105	0.141	0.753
98	T117	0.085	0.096	0.818
99	T118	0.267	0.083	0.650
100	T119	0.183	0.042	0.774
101	T141	0.164	0.150	0.687

102	T142	0.205	0.291	0.503
103	T155	0.312	0.074	0.614
104	T156	0.104	0.103	0.793
105	T120	0.817	0.108	0.076
106	T121	0.431	0.501	0.068
107	T122	0.797	0.149	0.054
108	T123	0.151	0.241	0.609
109	T124	0.099	0.148	0.753
110	T125	0.842	0.086	0.072
111	T126	0.313	0.583	0.105
112	T127	0.874	0.072	0.054
113	T131	0.472	0.458	0.070
114	T132	0.591	0.234	0.175
