# Supplementary material for: Kazakh national dog breed Tazy: What do we know?
Source: PLoS One. 2023 Mar 8;18(3):e0282041. doi: 10.1371/journal.pone.0282041 (PMC9994743; doi:10.1371/journal.pone.0282041)
Supplement: S5 Table — (PDF) [file pone.0282041.s006.pdf]

**S5 Table.** Sighthound dogs and cluster assignment.

| N | Label | Breed | Cluster1 | Cluster2 | Cluster3 | Cluster4 | Cluster5 |
|---|---|---|---|---|---|---|---|
| 1 | T12 | Tazy | **0.965** | 0.003 | 0.005 | 0.003 | 0.023 |
| 2 | T15 | Tazy | **0.969** | 0.008 | 0.006 | 0.004 | 0.013 |
| 3 | T26 | Tazy | **0.964** | 0.003 | 0.013 | 0.010 | 0.011 |
| 4 | T27 | Tazy | **0.960** | 0.003 | 0.015 | 0.016 | 0.007 |
| 5 | T50 | Tazy | **0.802** | 0.010 | 0.172 | 0.010 | 0.006 |
| 6 | T57 | Tazy | **0.977** | 0.009 | 0.005 | 0.005 | 0.004 |
| 7 | T59 | Tazy | **0.830** | 0.004 | 0.155 | 0.007 | 0.004 |
| 8 | T74 | Tazy | **0.962** | 0.004 | 0.004 | 0.027 | 0.004 |
| 9 | T88 | Tazy | **0.932** | 0.006 | 0.007 | 0.013 | 0.042 |
| 10 | T98 | Tazy | **0.980** | 0.003 | 0.007 | 0.005 | 0.005 |
| 11 | T105 | Tazy | **0.940** | 0.003 | 0.013 | 0.007 | 0.037 |
| 12 | T114 | Tazy | **0.967** | 0.008 | 0.014 | 0.004 | 0.007 |
| 13 | T118 | Tazy | **0.919** | 0.021 | 0.003 | 0.004 | 0.054 |
| 14 | W134 | Whippet | 0.008 | 0.003 | 0.004 | **0.983** | 0.003 |
| 15 | W135 | Whippet | 0.007 | 0.063 | 0.006 | **0.921** | 0.003 |
| 16 | W1 | Whippet | 0.017 | 0.009 | 0.010 | **0.948** | 0.016 |
| 17 | W2 | Whippet | 0.003 | 0.002 | 0.003 | **0.989** | 0.003 |
| 18 | W4 | Whippet | 0.009 | 0.012 | 0.007 | **0.965** | 0.007 |
| 19 | RB1 | Russian Borzoi | 0.011 | 0.018 | 0.008 | 0.003 | **0.960** |
| 20 | RB2 | Russian Borzoi | 0.005 | 0.006 | 0.002 | 0.003 | **0.984** |
| 21 | RB3 | Russian Borzoi | 0.240 | 0.016 | 0.013 | 0.032 | **0.699** |
| 22 | RB4 | Russian Borzoi | 0.005 | 0.008 | 0.004 | 0.003 | **0.980** |
| 23 | G1 | Greyhound | 0.109 | **0.525** | 0.068 | 0.213 | 0.084 |
| 24 | G2 | Greyhound | 0.068 | **0.881** | 0.009 | 0.036 | 0.006 |
| 25 | G3 | Greyhound | 0.003 | **0.990** | 0.004 | 0.002 | 0.002 |
| 26 | G4 | Greyhound | 0.027 | **0.739** | 0.005 | 0.031 | 0.198 |
| 27 | G5 | Greyhound | 0.005 | **0.979** | 0.003 | 0.004 | 0.009 |
| 28 | G6 | Greyhound | 0.004 | **0.989** | 0.002 | 0.003 | 0.002 |
| 29 | G8 | Greyhound | 0.010 | **0.977** | 0.007 | 0.004 | 0.003 |
| 30 | G7 | Greyhound | 0.009 | **0.974** | 0.004 | 0.003 | 0.010 |
| 31 | AH1 | Afghan Hound | 0.008 | 0.004 | **0.980** | 0.004 | 0.005 |
| 32 | AH2 | Afghan Hound | 0.006 | 0.005 | **0.978** | 0.005 | 0.005 |
| 33 | S1 | Saluki | **0.962** | 0.013 | 0.014 | 0.009 | 0.002 |
